# High expression of hypoxia inducible factor 1α related with acquired resistant to EGFR tyrosine kinase inhibitors in NSCLC

**DOI:** 10.1038/s41598-020-79801-1

**Published:** 2021-01-13

**Authors:** Qian Jin, Feihua Huang, Xianrong Xu, Haidong He, Yingqing Zhang

**Affiliations:** 1 Department of Respiratory Medicine, Tongde Hospital of Zhejiang Province, Hangzhou, Zhejiang 310012 People’s Republic of China; 2 Department of Respiratory Medicine, The First Hospital of Jiaxing, No. 1882 South Zhonghuan Road, Jiaxing, Zhejiang 314000 People’s Republic of China

**Keywords:** Cancer, Oncology

## Abstract

The acquired resistance of the first generation epidermal growth factor receptor-tyrosine kinase inhibitors (EGFR-TKIs) is a main factor leading to poor prognosis of non-small cell lung cancer (NSCLC), so we researched whether the high expression of hypoxia-inducible factor-1α (HIF-1α) in EGFR-TKIs sensitive NSCLC tissue tends to induce the acquired resistance. We detected the HIF-1α in normal lung tissue, EGFR-TKIs sensitive NSCLC tissue, the first generation EGFR-TKIs acquired resistant NSCLC tissue and acquired EGFR T790M mutation NSCLC tissue with the method of immunohistochemistry. Then, we compared the expression of HIF-1α in these tissues, and evaluate the effect of HIF-1α expression to the occurrence of acquired resistance. The expression of HIF-1α was much higher in the EGFR-TKIs sensitive NSCLC tissue than that in normal lung tissue. HIF-1α level became higher after the occurrence acquired resistance. There was negative correlation between HIF-1α level before receiving treatment and the time of acquired resistance occurring as well as the acquired EGFR T790M mutation occurring. As the treatment going on, EGFR-TKIs sensitivity rate of low HIF-1α level group was much higher than that of high level group. The high expression of HIF-1α related with the acquired resistance of the first generation EGFR-TKIs, and HIF-1α can be a biomarker to predict the early occurrence of acquired resistance.

## Introduction

In clinical treatment of non-small cell lung cancer (NSCLC), the acquired resistance of the first generation epidermal growth factor receptor-tyrosine kinase inhibitors (EGFR-TKIs) is a main factor leading to poor prognosis of NSCLC^1^. Hypoxia is an important character of solid tumors. Compared with tumors in oxygen-rich condition, tumors in hypoxic condition are more resistant to anti-tumor therapy, more invasive, more instable of genetic substance, more resistant to apoptosis and more potential for metastasis^2^. The mechanism of these effects refers to hypoxia-inducible factors (HIFs), especially HIF-1^3^.

HIF-1 signal pathway is the most important signal pathway activated in the hypoxic condition. HIF-1 contain α submit and β submit. The α protein, as the functional part of HIF-1, is oxygen sensitive submit expressing in hypoxic condition. The β submit is identical to aryl hydrocarbon nuclear translocator. There are more than one hundred downstream genes activated by HIF-1 signal pathway. In order to adapt the tumor’s need of survival and proliferation, proteins encoded by these genes participate in vascular growth of tumor, cell proliferation, survival, invasion and therapy resistance^4–6^. In these genes, MDR1, MET and ATP binding cassette transporter G2 gene are related with the therapy resistance of tumor^7^.

Previous researches indicated that the activation of HIF-1 signal pathway may induce the acquired resistance of other solid tumors to targeted therapy^8–15^. In EGFR-TKIs acquired resistant NSCLC cell lines, the expression of HIF-1α was much higher than that in EGFR-TKIs sensitive cell lines^16^. In NSCLC cell line with obvious effect to targeted therapy, the level of HIF-1α was significantly decreased, vice versa. Through artificially transfecting mutant with HIF-1α constitutive expression to NSCLC cell lines, the expression of HIF-1α became much higher, and the resistance of NSCLC to cetuximab was induced^17^.

Therefore, it has significance to research the effect of HIF-1 signal pathway in the acquired resistance of NSCLC target therapy. Clinical researches seldom detect the expression of HIF-1α in EGFR-TKIs sensitive and acquired resistant human NSCLC tissue. Thus, we designed this research, and hoped to identify the expression and clinical significance of HIF-1α in the first generation EGFR-TKIs sensitive and acquired resistant NSCLC.

## Results

### Study characteristics

A total of 233 EGFR sensitive mutation NSCLC patients and 96 controls were included in the study. 103 EGFR sensitive mutation NSCLC patients received treatment of the first generation EGFR-TKIs and were followed up for 30 months. Within the follow up period, none of the patients was lost to follow-up. But one patient died of acute myocardial infarction at the time of 8.1 month and one died of cerebrovascular accident at the time of 5.6 month. Related data were considered as censored data. In patients with EGFR-TKIs acquired resistance occurring in 30 months, totally 41 patients received a second biopsy and EGFR gene mutation detection showed that 20 patients were with acquired EGFR T790M mutation. EGFR-TKIs acquired resistant NSCLC tissues were collected. Demographics and clinical characteristics of these participants were shown in Table 1. EGFR sensitive mutation group and control group had no differences on age, sex and smoking.

**Table 1:** The clinical characteristics of EGFR sensitive mutation group, control group and EGFR-TKIs treatment group.

Category	Control group	EGFR sensitive mutation group	EGFR-TKIs treatment group	*P* value
**Subjects (n)**	96	233	103	
**Age (years, median (P25th, P75th))**	68 (63, 73)	69 (59, 76)	69 (57, 76)	0.287
**Gender**				0.336
Male (n, %)	43 (44.8)	91 (39.1)	58 (56.3)	
Femal (n, %)	53 (55.2)	142 (60.9)	45 (43.7)	
**Smoking**				0.870
Never (n, %)	63 (65.6)	150 (64.4)	53 (51.5)	
Ex and current (n, %)	33 (34.4)	83 (35.6)	50 (48.5)	
**Clinical stage**				
I–IIIA (n, %)		130 (55.8)	0 (0)	
IIIB–IV (n, %)		103 (44.2)	103 (100)	
**Pathological type**				
Squamous cell carcinoma (n, %)		12 (5.2)	5 (4.9)	
Adenocarcinoma (n, %)		220 (94.4)	97 (94.2)	
Adeno-squamous carcinoma (n, %)		1 (0.4)	1 (1.0)	
**EGFR sensitive mutation type**				
19del mutation (n, %)		103 (44.2)	51 (49.5)	
L858R mutation (n, %)		122 (52.4)	49 (47.6)	
Rare mutation (n, %)		8 (3.4)	3 (2.9)	
**EGFR-TKIs**				
Gefitinib (n, %)			31 (30.1)	
Erlotinib (n, %)			5 (4.9)	
Icotinib (n, %)			67 (65)	

*P* value for comparison between EGFR sensitive mutation group and control group.

### The tissue HIF-1α expression through immunohistochemical method

**Figure 1:**
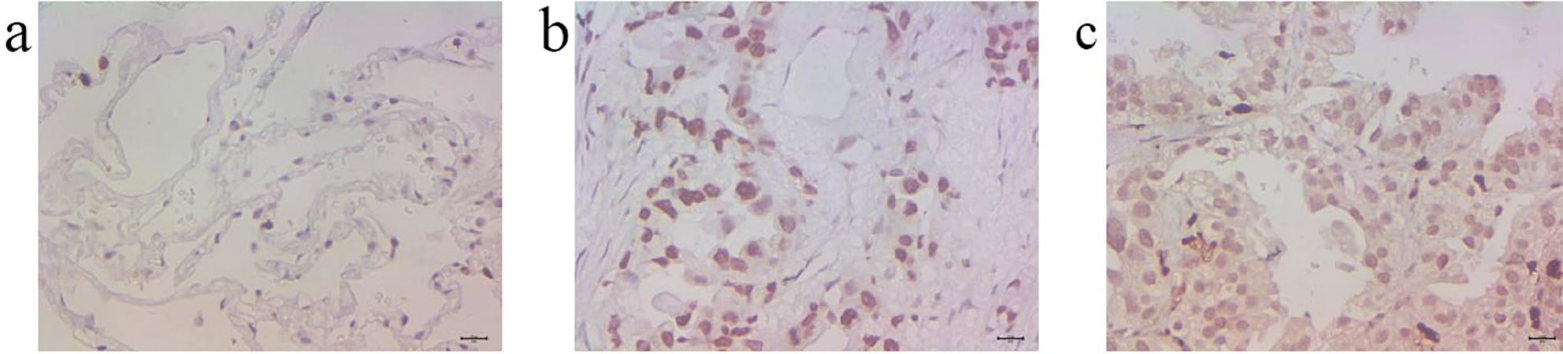
The tissue HIF-1 expression. (**a**) The negative HIF-1α expression of a normal lung tissue through immunohistochemical method (× 400). (**b**) The moderately positive HIF-1α expression of an EGFR-TKIs sensitive mutation NSCLC tissue through immunohistochemical method (× 400). (**c**) The strongly positive HIF-1α expression of EGFR-TKIs acquired resistant NSCLC tissue through immunohistochemical method (× 400).

The typical pictures of tissue HIF-1α expression were shown in Fig. 1. Immunohistochemical method was used to test the expression of HIF-1α in different groups. Semiquantitative scoring method was used to calculate the result. The scores of different groups were compared with Wilcoxon-Mann–Whitney test. HIF-1α level of EGFR sensitive mutation group was much higher than that of control group (*P* = 0.000). HIF-1α level of EGFR-TKIs treatment group was also higher than that of control group (*P* = 0.000). These results were shown in Table 2. For 41 cases receiving a second biopsy after gaining EGFR-TKIs acquired resistance in 30 months, the expression level of HIF-1α after acquired resistance in NSCLC tissue was higher than that before receiving EGFR-TKIs treatment (*P* = 0.000, Table 3). In these 41 patients, the expression level of HIF-1α after acquired resistance was higher than that before receiving EGFR-TKIs treatment for both T790M mutation group and without T790M mutation group (*P* = 0.012 and 0.001, respectively, Table 3). In 41 patients receiving a second biopsy, there were 31 (75.6%) patients had elevated HIF-1α expression after acquired resistance compared with the expression before treatment. For 31 patients with elevated HIF-1α expression and 10 patients with no HIF-1α expression elevating after acquired resistance, demographics and clinical characteristics were compared for them to find out whether patients with certain characteristic were tend to have HIF-1α elevating after EGFR-TKIs treatment, but the statistical analysis showed there was no characteristics difference between two groups (all *P* > 0.05, Table 4).

**Table 2:** The expression of HIF-1α in EGFR sensitive mutation group, EGFR-TKIs treatment group and control group.

Group	HIF-1α expression score Median (P25th, P75th)	*P* value
EGFR sensitive mutation group (n = 233)	6 (3, 8)	< 0.000*
EGFR-TKIs treatment group (n = 103)	6 (3, 9)	< 0.000*
Control group (n = 96)	2 (1, 3)	

*Compare with control group.

**Table 3:** The expression of HIF-1α in NSCLC tissues before receiving EGFR-TKIs treatment and after gaining EGFR-TKIs acquired resistance for patients receiving a second biopsy.

Group	HIF-1α expression score	Median (P25th, P75th)	*P* value
	Before EGFR-TKIs treatment	After EGFR-TKIs acquired resistance	
Second biopsy group (n = 41)	6 (3, 8.5)	8 (6, 10.5)	0.000
T790M mutation group (n = 20)	5.5 (2.25, 8.5)	7.5 (5.25, 9.75)	0.012
Without T790M mutation (n = 21)	7 (3, 8.5)	9 (8, 11)	0.001

**Table 4:** The clinical characteristics comparison of elevated HIF-1α group and no HIF-1α elevating group in 41 patients receiving a second biopsy.

Category	Elevated HIF-1α group	No HIF-1α elevating group	*P* value
**Subjects (n)**	31	10	
**Age (years, median (P25th, P75th))**	68 (55, 75)	71 (68.25, 74.5)	0.224
**Gender**			0.414
Male (n, %)	17 (54.8)	4 (40)	
Femal (n, %)	14 (45.2)	6 (60)	
**Smoking**			0.379
Never (n, %)	17 (54.8)	7 (70)	
Ex and current (n, %)	14 (45.2)	3 (30)	
**Clinical stage**			
IIIB–IIIC (n, %)	10 (32.3)	6 (60)	0.118
IVA–IVB (n, %)	21 (67.7)	4 (40)	
**Pathological type**			
Squamous cell carcinoma (n, %)	2 (6.5)	0 (0)	0.793
Adenocarcinoma (n, %)	29 (93.5)	10 (100)	
**EGFR sensitive mutation type**			
19del mutation (n, %)	16 (51.6)	4 (10)	0.523
L858R mutation (n, %)	15 (48.4)	6 (60)	
**EGFR-TKIs**			
Gefitinib (n, %)	10 (32.3)	2 (20)	0.459
Icotinib (n, %)	21 (67.7)	8 (80)	

### Relationship between HIF-1α expression and tumor stage and correlation between HIF-1α expression and tumor diameter

Kruskal–Wallis rank sum test was used to analyze the difference of HIF-1α expression among clinical tumor stages. For EGFR sensitive mutation cases, EGFR-TKIs treatment cases, receiving second biopsy cases and T790M mutation cases (both HIF-1α level before treatment and after acquired resistance were analyzed for second biopsy cases and T790M mutation cases), there was no different HIF-1α expression among tumor stages (*P* = 0.320, 0.137, 0.510, 0.216, 0.547 and 0.207, respectively, Table 5). Pearson's correlation analysis also showed that there was no correlation between HIF-1α expression and tumor stage in various groups (all *P* > 0.05 and all R^2^ < 0.3, Table 5). For correlation between HIF-1α expression and tumor diameter of biopsy site (diameter was the average of long and short diameter) in various groups, Pearson’s correlation analysis showed no significant correlation (all *P* > 0.05, and all R^2^ < 0.3 except R^2^ = 0.469 of EGFR sensitive mutation group, Table 6).

**Table 5:** Relationship analysis between HIF-1α level and clinical stage through HIF-1α level comparison analysis among stages and correlation analysis between stage and HIF-1α level.

Group	Comparison analysis	Correlation analysis	
	*P* value	*P* value	R^2^ value
EGFR sensitive mutation group (n = 233)	0.320	0.131	0.010
EGFR-TKIs treatment group (n = 103)	0.137	0.482	0.005
Second biopsy group before treatment (n = 41)	0.510	0.244	0.035
Second biopsy group after acquired resistance (n = 41)	0.216	0.087	0.073
T790M mutation group before treatment (n = 20)	0.547	0.559	0.019
T790M mutation group after acquired resistance (n = 20)	0.207	0.146	0.114

**Table 6:** Pearson's correlation analysis between tumor diameter and HIF-1α level.

Group	*P* value	R^2^ value
EGFR sensitive mutation group (n = 233)	0.056	0.469
EGFR-TKIs treatment group (n = 103)	0.982	0.292
Second biopsy group before treatment (n = 41)	0.754	0.183
Second biopsy group after acquired resistance (n = 41)	0.551	0.278
T790M mutation group before treatment (n = 20)	0.194	0.047
T790M mutation group after acquired resistance (n = 20)	0.218	0.165

### Correlation between HIF-1α expression and EGFR-TKIs acquired resistance

**Figure 2:**
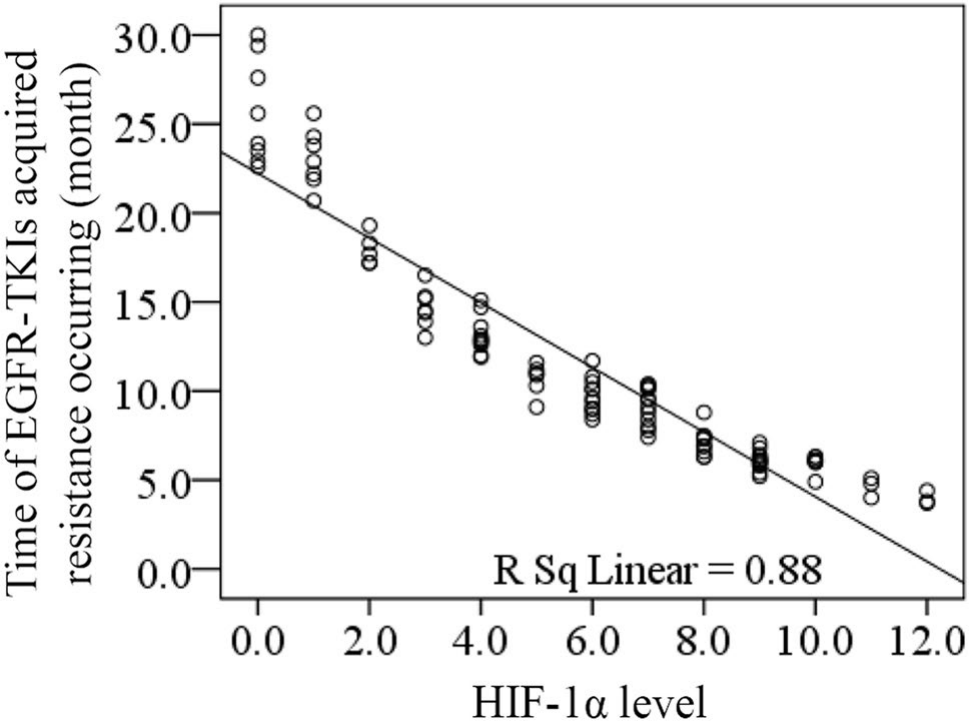
The correlation between the HIF-1α level before receiving treatment and the time of EGFR-TKIs acquired resistance occurring for 103 cases receiving EGFR-TKIs treatment. Linear correlation analysis was adopted to evaluate the correlation between the HIF-1α level before receiving treatment and the time of EGFR-TKIs acquired resistance occurring, and the result showed the negative correlation (R = − 0.938, *P* = 0.000).

**Figure 3:**
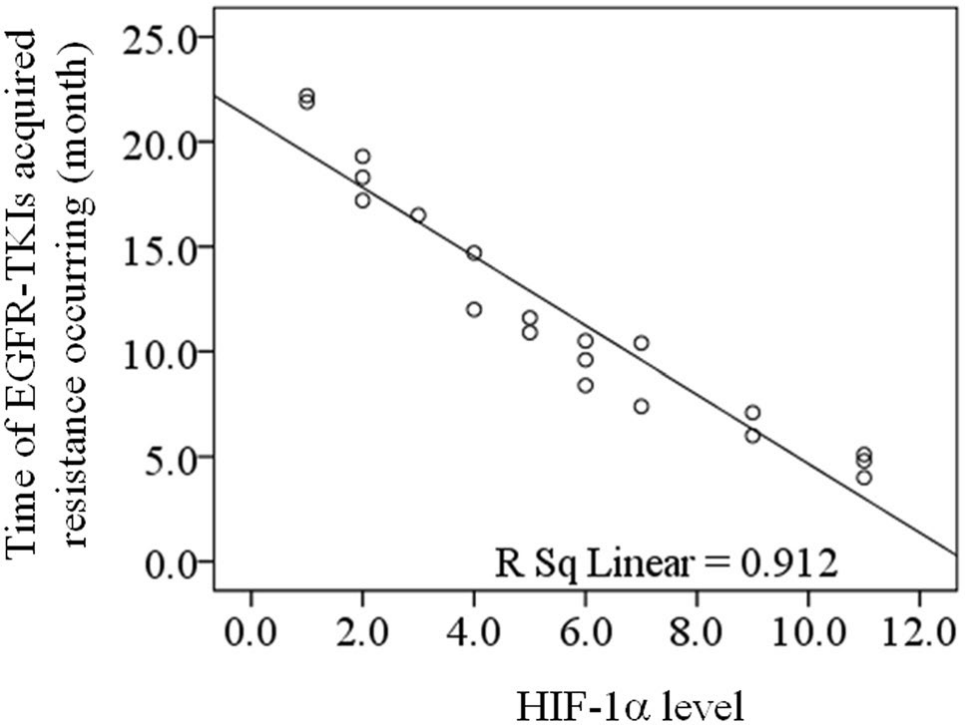
The correlation between the HIF-1α level before receiving treatment and the time of EGFR-TKIs acquired resistance occurring for 20 cases with acquired EGFR T790M mutation. Linear correlation analysis was adopted to evaluate the correlation between the HIF-1α level before receiving treatment and the time of EGFR-TKIs acquired resistance occurring, and the result showed the negative correlation (R = − 0.955, *P* = 0.000).

**Figure 4:**
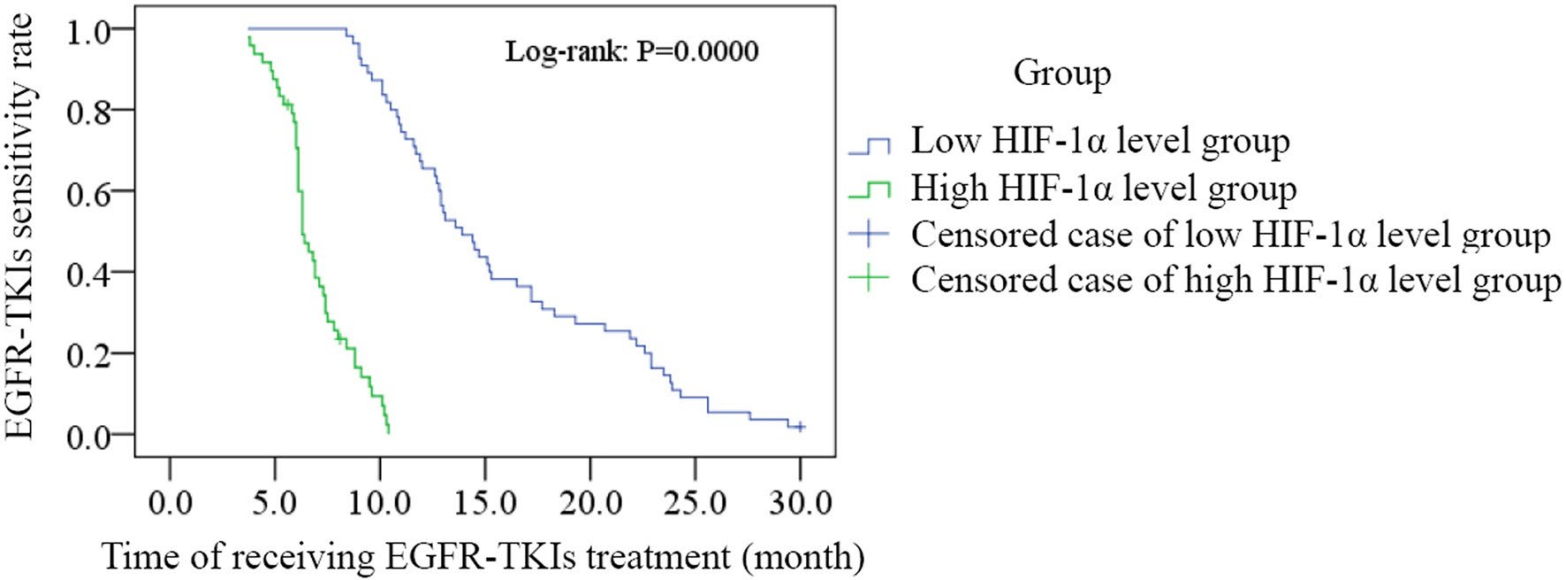
Kaplan–Meier analysis evaluated the EGFR-TKIs sensitivity rate of low HIF-1α level group and high HIF-1α level group in 30 months for 103 cases receiving EGFR-TKIs treatment**.** 103 EGFR-TKI sensitive mutation patients were divided into low HIF-1α level group (n = 55, ≤ median) and high HIF-1α level group (n = 48, > median) according to the median level of HIF-1α before receiving treatment. Vertical bars indicate cases with censored data. Kaplan–Meier survival analysis was conducted. As the treatment going on, EGFR-TKIs sensitivity rate of low HIF-1α level group was much higher than that of high HIF-1α level group (log-rank: *P* = 0.000).

**Figure 5:**
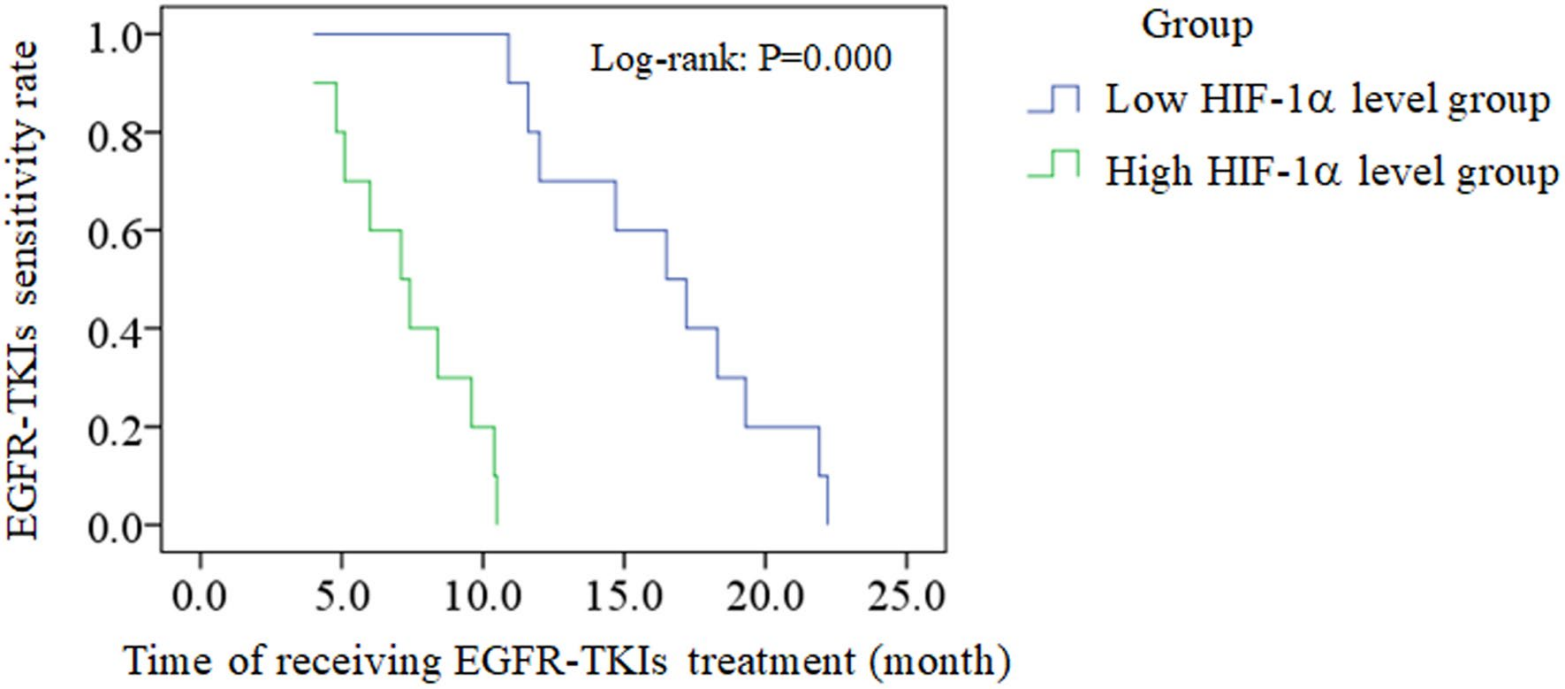
Kaplan–Meier analysis evaluated the EGFR-TKIs sensitivity rate of low HIF-1α level group and high HIF-1α level group in 30 months for 20 cases with acquired EGFR T790M mutation**.** 20 acquired EGFR T790M mutation patients were divided into low HIF-1α level group (n = 10, ≤ median) and high HIF-1α level group (n = 10, > median) according to the median level (median = 5.5) of HIF-1α before receiving treatment. Kaplan–Meier survival analysis was conducted. EGFR-TKIs acquired resistance happened in a much shorter time of high HIF-1α level group than that of low HIF-1α level group (log-rank: *P* = 0.000).

For 103 cases receiving EGFR-TKIs treatment and 20 cases with acquired EGFR T790M mutation, linear correlation analysis was adopted to evaluate the correlation between the HIF-1α level before receiving treatment and the time of EGFR-TKIs acquired resistance occurring, and both results showed the negative correlation (R = − 0.938, *P* = 0.000, Fig. 2; R = − 0.955, *P* = 0.000, Fig. 3). According to the median level of HIF-1α in 103 EGFR-TKI sensitive mutation patients before receiving treatment (median = 6), 103 patients were divided into low HIF-1α level group (n = 55, ≤ median) and high HIF-1α level group (n = 48, > median). Vertical bars indicate cases with censored data (including non-NSCLC death cases and cases keeping sensitive to EGFR-TKIs in 30 months EGFR-TKIs treatment). Then Kaplan–Meier survival analysis was conducted. As the treatment going on, EGFR-TKIs sensitivity rate of low HIF-1α level group was much higher than that of high HIF-1α level group (log-rank: *P* = 0.000, Fig. 4). For 20 cases with acquired EGFR T790M mutation, this Kaplan–Meier survival analysis was also performed, and EGFR-TKIs acquired resistance happened in a much shorter time of high HIF-1α level group than that of low HIF-1α level group (log-rank: *P* = 0.000, Fig. 5). For 103 cases receiving EGFR-TKIs treatment, clinical characteristics were analyzed by multivariate Cox regression analysis to identify if they were influencing factors of EGFR-TKIs acquired resistance occurring. During these clinical characteristics, only HIF-1α level and EGFR-TKI type had statistical significance (*P* = 0.000 and *P* = 0.007, respectively). Then Kaplan–Meier survival curves were drawn for the clinical characteristics of EGFR-TKI type, and we found that the curves were crossed. At the same time, only 5 patients received erlotinib treatment in our study, so it had no significance to point out which EGFR-TKI had worse outcome. Thus EGFR-TKI type was not an influencing factors of EGFR-TKIs acquired resistance occurring. For the clinical characteristics of HIF-1α level, the high HIF-1α level before treatment was able to cause a low EGFR-TKIs sensitivity rate after a period of EGFR-TKIs treatment. It is a significant negative influencing factor (*P* = 0.000, HR = 0.045, Table 7). For 20 cases with acquired EGFR T790M mutation, clinical characteristics were also analyzed by multivariate Cox regression analysis, and only HIF-1α level had statistical significance (*P* = 0.042, HR = 0.004, Table 8).

**Table 7:** Cox regression analysis of clinical characteristics as influencing factors of EGFR-TKIs acquired resistance occurring for 103 cases receiving EGFR-TKIs treatment.

Clinical characteristics	HR	95% CI	*P* value
Age	0.925	0.591–1.447	0.731
Gender	0.653	0.351–1.215	0.178
Smoking	0.649	0.348–1.207	0.172
Clinical stage	1.016	0.658–1.569	0.944
EGFR sensitive mutation type	0.874	0.592–1.291	0.498
EGFR-TKI type	0.722	0.570–0.915	0.007
HIF-1α level	0.045	0.021–0.099	0.000

*HR* hazard ratio, *CI* confidence interval.

**Table 8:** Cox regression analysis of clinical characteristics as influencing factors of EGFR-TKIs acquired resistance occurring for 20 cases with acquired EGFR T790M mutation.

Clinical characteristics	HR	95% CI	*P* value
Age	0.984	0.125–7.738	0.987
Gender	1.098	0.443–2.718	0.840
Smoking	0.791	0.297–2.105	0. 639
Clinical stage	0.922	0.243–3.501	0.905
EGFR sensitive mutation type	0.869	0.347–2.173	0.764
EGFR-TKI type	0.825	0.278–2.452	0.729
HIF-1α level	0.004	0.000–1.324	0.042

*HR* hazard ratio, *CI* confidence interval.

## Discussion

EGFR-TKIs have developed from the first generation to the third generation. The first generation EGFR-TKIs is more efficient and more affordable, and it remains as the main target therapy medication for NSCLC with advanced stage. Therefore, it is with significance to research the acquired resistance of the first generation EGFR-TKIs. Previous researches have indicated the sensitivity influence of HIF-1α on target therapy of other solid tumors^9–15^. Nevertheless, the influence of HIF-1α on EGFR target therapy of NSCLC needs further and more researches.

In our study, the human EGFR gene 29 mutation fluorescence PCR diagnostic kit has excellent sensitivity and specificity. It can also detect the rare mutation of EGFR. In previous researches, the sensitivity of the first generation EGFR-TKIs to the rare mutation of EGFR in NSCLC kept discordant^20,21^. The multivariate Cox regression analysis identified that the type of EGFR mutation was not the influencing factor of the acquired resistance of the first generation EGFR-TKIs.

In EGFR-TKIs sensitive NSCLC tissue, the expression of HIF-1α was higher than that in normal lung tissue. This was accordant with previous researches^16,17,22–24^. NSCLC is a kind of solid tumor, and hypoxia is one of its characteristics. NSCLC cells rapidly proliferated and formed a large size in a short time. Because of the compression in the solid tumor, vessels were obstructed and hypoxia was present in the center area^25^. Cancer cells in hypoxic condition activate related pathways to adapt the hypoxia, and the elevated HIF-1α level was the indication of HIF-1α signal pathway activation^26^.

We also tested the HIF-1 level of NSCLC tissue after the resistance occurred. We found that the expression of HIF-1α was much higher in EGFR-TKIs acquired resistant NSCLC tissues than that in EGFR-TKIs sensitive NSCLC tissues before receiving the first generation EGFR-TKIs treatment. This result was accordant with previous research^17^. But this result can’t clarify causation. So we still can’t identify that it is the acquired resistance of the first generation EGFR-TKIs caused the elevated level of HIF-1α in NSCLC tissue or the up-regulation of HIF-1 signal pathway induced the acquired resistance. Thus we conducted Kaplan–Meier survival analysis to further clarify the causation.

In our study, 103 EGFR sensitive mutation NSCLC patients were divided into low HIF-1α group and high HIF-1α group according to the expression level of HIF-1α in NSCLC tissues before receiving EGFR-TKIs treatment. Kaplan–Meier survival analysis was conducted base on this grouping. Through this analysis, we found that EGFR-TKIs sensitive rate of low HIF-1α group became higher than that of high HIF-1α group as therapy time passed. Patients of high HIF-1α group can present EGFR-TKIs acquired resistance in a short time. This result was accordant with a previous research for cetuximab acquired resistance^17^. Therefore, we concluded that high expression of HIF-1α before receiving the first generation EGFR-TKIs therapy was able to induce the acquired resistance of the first generation EGFR-TKIs. A series of previous researches indicated that the activation of HIF-1 signal pathway induced the resistance of solid tumor to medication therapy. HIF-1 pathway achieved this effect through many mechanisms. First, HIF-1 signal pathway changed the cell metabolism to adapt the hypoxia condition^27–30^. Second, hypoxia disturbed the cell apoptosis and autophagy^31–33^. Third, hypoxia led to genome instabilitiy^34–37^. Fourth, hypoxia can induce epithelial-to-mesenchymal transition (EMT)^38–43^. Last, hypoxia promotes the invasiveness and metastasis of tumor cells by regulating extracellular matrix dynamics^44–49^.

There are many confounding factors in clinical studies. Except the analysis for differences of age, gender and smoking in different groups, we conducted Cox regression analysis to identify if age, gender, smoking, clinical stage, EGFR sensitive mutation type, EGFR-TKIs type and HIF-1α level were influencing factors of EGFR-TKIs acquired resistance occurring. Finally, only the *P* value of HIF-1α level was less than 0.0001, and the value of HR was 0.045 which was much less than 1. This indicated that HIF-1α level was the influencing factor of EGFR-TKIs acquired resistance occurring. Further, previous researches reported that HIF-1α may relate with tumor stage and tumor size^50^. In our study, we analyzed the relationship between HIF-1α level and clinical stage, as well as correlation between HIF-1α level and tumor diameter of biopsy site. However, our results for different groups didn’t show obviously correlations. Thus, we didn’t think that tumor stage and tumor size were indirectly related with the acquired EGFR-TKIs resistance according to the present data. For EGFR sensitive mutation group containing 233 cases, the *P* value and R^2^ value of correlation analysis between tumor diameter and HIF-1α level were 0.056 and 0.469, so we can’t absolutely deny the correlation. Maybe a larger sample size is needed to obtain a statistically significant result. In addition, we found 31 patients with elevated HIF-1α expression after acquired EGFR-TKIs resistance tended to have advanced stage, though its *P* value was 0.118 while the sample size was very small (Table 4). It is possible that HIF-1α level in advanced stage NSCLC is easy to increase in the process of EGFR-TKIs treatment, and these patients can obtain more benefits from anti-HIF-1 therapy in the future. Further research with a large sample is necessary.

Our analysis results also suggested that HIF-1α might be the drug resistant factor for the first generation EGFR-TKIs. However, no previous researches focused on HIF-1α as an initial drug resistant factor of EGFR-TKIs. To clarify this, we reviewed the initial data of 103 patients receiving EGFR-TKIs treatment, and found that the earliest time of acquired EGFR-TKIs resistance occurring was 3.7 months with a high HIF-1α expression score of 12 before receiving EGFR-TKI treatment. At least in this 3.7 months, the patient was sensitive to EGFR-TKI. For other two patients with high HIF-1α expression score of 12 before receiving EGFR-TKIs treatment, their times of acquired EGFT-TKIs resistance occurring were 4.4 and 3.8 months respectively. This indicated that HIF-1α was not an initial drug resistant factor of the first generation EGFR-TKIs.

In acquired EGFR T790M mutation patients, HIF-1α level was elevated as well, and patients with high HIF-1α level before receiving EGFR-TKIs treatment can also present acquired EGFR T790M mutation in a shorter time. These results were obtained through a small sample size. It showed an obvious statistical significance. For other mechanisms of acquired EGFR-TKIs resistance such as MET amplification, we didn’t perform statistical analysis because of a very small sample size. However, there was no previous researches mentioned the correlation between HIF-1 and T790M mutation. This discovery of HIF-1α and acquired T790M mutation here deserves further research and potential mechanism research.

In conclusion, we identified the expression of HIF-1α in EGFR sensitive mutation NSCLC tissues, normal lung tissues and the first generation EGFR-TKIs acquired resistant NSCLC tissues. Through this clinical research, we also found that the expression level of HIF-1α was much higher after the acquired resistance occurred, and patients with high HIF-1α level before receiving EGFR-TKIs treatment can present EGFR-TKIs acquired resistance in a shorter time. These phenomena were also obvious in acquired EGFR T790M mutation patients. Thus, we concluded that the high expression of HIF-1α related with the acquired resistance of the first generation EGFR-TKIs, and HIF-1α can be a biomarker to predict the early occurrence of acquired resistance of the first generation EGFR-TKIs.

## Materials and methods

### Study participants

This study contained EGFR sensitive mutation group and control group. 233 EGFR sensitive mutation NSCLC patients and 96 controls with normal lung tissue were included in this study. Study participants were collected from the First Hospital of Jiaxing City and Tongde Hospital of Zhejiang Province between 06/2014 and 12/2017. EGFR sensitive mutation patients met the following inclusion criteria: (1) NSCLC patients with definite pathologic diagnosis. (2) With EGFR sensitive mutation. (3) Without other malignancies. (4) Asiatic race between 18 and 80 years old. Control group met the following inclusion criteria: (1) Performing bronchoscopy or lobectomy/segmentectomy because of lung nodule which was identified as pulmonary fibroproliferative nodule after operation. (2) Without malignancy. (3) Asiatic race between 18 and 80 years old. Exclusion criteria of participants: (1) Not Asiatic race. (2) Older than 80 years old or younger than 18 years old. (3) With malignancy except NSCLC. Withdrawal criteria: (1) Diagnosed with other malignancy in 30 months after included in study. (2) Died in 3 months after diagnosed with NSCLC.

### Study design

For control group, the normal lung tissue near to the nodule was collected. For EGFR sensitive mutation group, NSCLC tissue was obtained through surgery, bronchoscopy and percutaneous lung biopsy. We took NSCLC tissues from the primary foci and avoiding the necrotic area. In EGFR sensitive mutation group, patients receiving EGFR-TKIs treatment were defined as EGFR-TKIs treatment group and were designed to follow up for 30 months. Follow-up was performed every month. We recorded when the EGFR-TKIs acquired resistance occurred. EGFR-TKIs acquired resistance was defined as the end point. For patients occurred EGFR-TKIs acquired resistance in 30 months, a second biopsy was recommeded to obtain EGFR-TKIs acquired resistant NSCLC tissue. In this study, we tested and compared the level of HIF-1α in tissues of two groups through immunohistochemical method. We also tested the level of HIF-1α in the tissues of NSCLC patients after the occurrence of EGFR-TKIs acquired resistance, in order to compare it with that before receiving EGFR-TKIs treatment. Through the data from follow-up, we analyzed the relationship between HIF-1α level and the time of EGFR-TKIs acquired resistance occurring.

### Detection of EGFR gene mutation

NSCLC tissues obtained through surgery, bronchoscopy and percutaneous lung biopsy were collected and fixed in 10% neutral formalin fix solution for 48 h. After paraffin embedding, 5 μm sections were prepared. DNA of NSCLC tissue was extracted according to the instruction of DNA extraction Kit (Qiagen GmbH, Hilden, Germany). Human EGFR gene 29 mutation fluorescence polymerase chain reaction (PCR) diagnostic kit (Amoy Diagnostics Co.,Ltd., Xiamen, China) was used to detect the EGFR gene mutation in these NSCLC specimens with the method of real-time PCR. DNA concentration was adjusted to 3 ng/μl. 2.7 μl Taq polymerase was added into 42.3 μl DNA sample, positive control and purity water. 5 μl of these samples were added into 8-tube strip and centrifuged at 8000×*g* for 10 s, then putted into Applied Biosystems 7500 Real-Time PCR system (Applied Biosystems, Foster City, CA, USA) for detection. The primer sequences of EGFR 18–21 exons were shown in Table 9. EGFR gene amplification was designed as the following procedure: 95 ℃ for 5 min, 1 cycle; 95 ℃ for 25 s, 64 ℃ for 20 s, 72 ℃ for 20 s, totally 15 cycles; 93 ℃ for 25 s, 60 ℃ for 35 s, 72 ℃ for 20 s, totally 31 cycles.

**Table 9:** The primer sequences of EGFR 18–21 exons.

Amplification site	Primer	Sequence
18 exon	Forward primer	TGTCCTGGCACCCAAGCCCATGCCGTGGCT
	Reverse primer	GTGGGGAGCCCAGAGTCCTTGCAAGCTGTATA
19 exon	Forward primer	CAGTGTCCCTCACCTTCGGGGTGCATCGCT
	Reverse primer	AACCTCAGGCCCACCTTTTCTCATGTCTGG
20 exon	Forward primer	GCCCTGCGTAAACGTCCCTGTGCTAGGTCT
	Reverse primer	CACATGCGGTCTGCGCTCCTGGGATAGCAA
21 exon	Forward primer	CCATTCTTTGGATCAGTAGTCACTAACGT
	Reverse primer	CCAGGCTGCCTTCCCACTAGCTGTATTG

The EGFR gene mutation result was judged by two professional analysts. If the analysts gave different judgment, a third analyst was invited to give a third judgment. The final result must be come from accordant judgment of two analysts.

### Detection of tissue HIF-1α expression through immunohistochemical method

The method of collection and fixation of NSCLC tissue and normal lung tissue was the same with the method used in the detection of EGFR gene mutation. Paraffin sections were treated with heat mediated antigen retrieval after deparaffinage and rehydration. Sections were put into citrate buffer solution. The temperature of citrate buffer solution ranged from 92℃ to 98℃ for 10–15 min by microwave heating method. Then citrate buffer solution was put in room temperature for 10–20 min and washed in PBS. Specimen sections were treated with immunohistochemical test according to the instruction of kit (Abcam, Cambridge, MA, USA). 0.5% TritonX-100 was added on sections at room temperature for 20 min and sections were washed in PBS for three times (3 min per time). Then sections were treated with 3% H_2_O_2_ at room temperature for 15 min and washed in PBS for three times (3 min per time). Tissues on sections were blocked by goat serum at room temperature for 60 min and then incubated with the rabbit polyclonal primary antibody (cat. no. ab231951; Abcam, Cambridge, MA, USA) overnight at 4 ℃ and at a dilution of 1:100. Negative control sections were incubated with non-immune rabbit serum instead of rabbit polyclonal primary antibody. Sections were washed in PBS for three times (5 min per time) and then incubated with the goat anti rabbit and horseradish peroxidase-labeled polyclonal secondary antibody (cat. no. ab205718; Abcam, Cambridge, MA, USA) at 37℃ for 30 min and at a dilution of 1:60. Sequentially, sections were washed in PBS for three times (5 min per time) and stained with 3,3′-diaminobenzidine for 3 to 10 min (avoid light, observe tissues under microscope until it turn to brown). After staining, sections were washed in distilled water for two times (5 min per time), re-stained with hematoxylin for 5 min, returned blue with tap water, dehydrated in a graded ethanol series (75%, 85%, 95% and 100% ethanol, 3 min for each concentration) and cleared in xylene for two times (3 min per time). Finally, sections were enveloped with netural gum. Breast cancer tissue with known positive expression served as a positive control.

All sections were observed under a light microscope. The result of immunohistochemical test was judged by two professional analysts. If the analysts gave different judgment, a third analyst was invited to give a third judgment. The final result must be come from accordant judgment of two analysts. The result was calculated according to the semiquantitative scoring method^18,19^. Every section was observed for five high power fields randomly. Then the positive cell percentage and the positive expression intensity were recorded. The scoring criteria of the positive cell percentage: 0: Positive cell ≤ 5%; 1: Positive cell 6–25%; 2: Positive cell 26–50%; 3: Positive cell 51–75%; 4: Positive cell ≥ 76%. The scoring criteria of the positive expression intensity: 0: No colorization; 1: Yellow-colored; 2: Yellow brown-colored; 3: Dark brown-colored. Final result of HIF-1α immunohistochemical test was acquired from the product of positive cell score and positive intensity score: 0: Negative (−); 1–4: Weakly positive (+); 5–8: Moderately positive (++); 9–12: Strongly positive (+++).

### Statistical analysis

Statistical analysis was carried out utilizing SPSS software (version 16.0.0, SPSS, Inc., Chicago, IL, USA). Age of participants and expression levels of HIF-1α in different groups were compared using Wilcoxon-Mann–Whitney test. Sex, pathological type and smoking difference in groups were compared with Pearson's chi-squared test. The correlation analysis was performed with the Pearson's correlation analysis. HIF-1α level comparison between different clinical stages was analyzed with Kruskal–Wallis rank sum test. Patients receiving EGFR-TKIs treatment were followed up for 30 months, and the median of HIF-1α expression level was used as the cut-off value for dichotomizing HIF-1α expression. The time of EGFR-TKIs acquired resistance occurring was calculated from the date of collecting to the date of EGFR-TKIs acquired resistance occurring or loss to follow-up. Survival curves (event was defined as EGFR-TKIs acquired resistance occurring) were generated using the method of Kaplan–Meier. For the expression levels of HIF-1α in the tissues of NSCLC patients before receiving EGFR-TKIs treatment and after EGFR-TKIs acquired resistance occurring, Wilcoxon singed rank test of pair matching data was used to compare their difference. Clinical characteristics were analyzed by multivariate Cox regression analysis to identify if they were influencing factors of EGFR-TKIs acquired resistance occurring. A *P* value < 0.05 was considered statistically significant.

### Ethics approval and consent to participate

The study protocol was approved by the Ethical Committee of Tongde Hospital of Zhejiang Province (Q[2014]012) and the First Hospital of Jiaxing City (LS2014-284), and all procedures performed in studies involving human participants were in accordance with the 1964 Helsinki declaration and its later amendments or comparable ethical standards. Written informed consent to participate was obtained from all participants.

### Consent for publication

Written informed consent for the publication of any associated data and accompanying images was obtained from all participants.

## Data Availability

The datasets used and analyzed during this study are available from the corresponding author on reasonable request.
